# Mediation Analysis with Survival Outcomes: Accelerated Failure Time vs. Proportional Hazards Models

**DOI:** 10.3389/fpsyg.2016.00423

**Published:** 2016-03-30

**Authors:** Lois A. Gelfand, David P. MacKinnon, Robert J. DeRubeis, Amanda N. Baraldi

**Affiliations:** ^1^Department of Psychology, University of PennsylvaniaPhiladelphia, PA, USA; ^2^Department of Psychology, Arizona State UniversityTempe, AZ, USA; ^3^Department of Psychology, Oklahoma State UniversityStillwater, OK, USA

**Keywords:** survival analysis, statistical mediation, monte carlo simulation, potential outcomes, causal modeling

## Abstract

**Objective:** Survival time is an important type of outcome variable in treatment research. Currently, limited guidance is available regarding performing mediation analyses with survival outcomes, which generally do not have normally distributed errors, and contain unobserved (censored) events. We present considerations for choosing an approach, using a comparison of semi-parametric proportional hazards (PH) and fully parametric accelerated failure time (AFT) approaches for illustration.

**Method:** We compare PH and AFT models and procedures in their integration into mediation models and review their ability to produce coefficients that estimate causal effects. Using simulation studies modeling Weibull-distributed survival times, we compare statistical properties of mediation analyses incorporating PH and AFT approaches (employing SAS procedures PHREG and LIFEREG, respectively) under varied data conditions, some including censoring. A simulated data set illustrates the findings.

**Results:** AFT models integrate more easily than PH models into mediation models. Furthermore, mediation analyses incorporating LIFEREG produce coefficients that can estimate causal effects, and demonstrate superior statistical properties. Censoring introduces bias in the coefficient estimate representing the treatment effect on outcome—underestimation in LIFEREG, and overestimation in PHREG. With LIFEREG, this bias can be addressed using an alternative estimate obtained from combining other coefficients, whereas this is not possible with PHREG.

**Conclusions:** When Weibull assumptions are not violated, there are compelling advantages to using LIFEREG over PHREG for mediation analyses involving survival-time outcomes. Irrespective of the procedures used, the interpretation of coefficients, effects of censoring on coefficient estimates, and statistical properties should be taken into account when reporting results.

## Introduction

Treatment mediators are variables that transmit a treatment effect to an outcome variable. The promise of mediation analysis in treatment research is to identify underlying mechanisms by which treatment actions lead to beneficial outcomes, and to improve treatments by maximizing the activity of these mechanisms. Mediation analyses can provide useful information both when the expected treatment effect occurs and when it does not. In the latter situation, one can investigate whether the failure to find a treatment effect occurred because the treatment did not affect the mediator as intended (failure in “action” theory), or because the mediator was not associated with outcome as theorized (failure in “conceptual” theory; Chen, 1990; MacKinnon, 2008). Because of the benefits of identifying the mechanisms of actions via a mediator, the examination of potential mediators of treatment effects has generated substantial interest. In 2002, for example, Kraemer and colleagues recommended that all randomized controlled trials include a plan to perform mediation analyses to “narrow the search for causal mechanisms” of psychiatric treatments with the goal of refining theory and enhancing treatment effectiveness (Kraemer et al., 2002, p. 878). A growing number of treatment researchers have called for more attention to mediation to test the theory underlying treatments and to identify treatment actions that produce change in the mediator(s) and the outcomes (Kazdin, 2000; Weersing and Weisz, 2002; Kazdin and Nock, 2003; Nock, 2007; Longabaugh and Magill, 2011).

The procedures of mediation analysis depend in part on the nature of the independent variable, the potential mediating variables, and the dependent variables under investigation. Whereas mediation analysis with continuous mediators and outcomes via linear regression modeling has been widely discussed since Baron and Kenny's (1986) influential article, methods for performing mediation analysis with other types of data, such as dichotomous (MacKinnon and Dwyer, 1993; MacKinnon et al., 2007; VanderWeele and Vansteelandt, 2010) or longitudinal dependent variables (see MacKinnon, 2008 for review) are now receiving increasing attention. The variable, *event time*, where the measure is the time until an event occurs, is a type of outcome variable that is important in clinical treatment research, but one for which guidance for researchers interested in performing mediation analysis is still relatively limited. Event time outcomes common in clinical research include time to response (or remission or recovery) during treatment and time to relapse or recurrence (e.g., to depression or substance use) after treatment. Other examples include time to an adverse event (e.g., a switch to mania during treatment with antidepressant medication), and time to the occurrence of a behavior (e.g., re-offense after release from prison). “Event time” is alternatively referred to as “survival time” (from its use in biomedical contexts), or “failure time” (from its use in industrial contexts). In this paper, we will use event time, survival, and survival time interchangeably, and refer to procedures to analyze dependent variables of this type as survival analyses.

Survival outcomes in behavioral research generally include missing data because the event of interest is not observed for all subjects; such missing observations are conventionally referred to as “censored.” In this paper, survival outcomes that are censored for two reasons are considered: (1) study duration censoring occurs when an event is not observed for a participant because the event did not occur within the observation period for the study; or (2) dropout censoring occurs when the event is not observed because the participant was lost to the study before having experienced an event. It is assumed that the process of losing participants is unrelated to their event times; formally, study duration censoring is an example of Type I right censoring and dropout censoring is an example of random right censoring (Allison, 1995). The variable, event time, generally does not follow a normal distribution. The analysis of event times is relatively more complex than that of typical continuous variables, due to the presence of censored data, non-normal event time distributions, and the unfolding of events over time.

The semi-parametric Cox proportional hazards (PH) regression (Cox, 1972) has arguably become the default procedure for analyzing survival data in many behavioral research contexts (see Singer and Willett, 1993; Allison, 1995) due to relatively unrestrictive assumptions. In particular, the survival time does not need to follow any specific distribution. In a PH regression (e.g., the SAS PHREG procedure) the log of the hazard, which is a measure of the rate at which events occur (see Allison, 1995), serves as the dependent variable. PH models describe treatment effects in which the rate at which events occur to participants in one treatment group is a constant proportion (increased or decreased) of the rate in the other group. For example, patients who have recovered from depression and are exposed to a mindfulness treatment may relapse at half the rate of patients in a control condition in any given time period. There are similarities between PH regression and linear regression, but because there are issues specific to the PH model (as will be discussed later), these procedures cannot necessarily be applied in the same way to investigate mediation. One class of fully parametric survival models that, under certain distributional assumptions, serves as a potential alternative to the proportional hazards model is the accelerated failure time (AFT) model. The AFT model treats the log-survival time rather than the log hazard as the dependent variable in a parametric survival regression (e.g., via the SAS LIFEREG procedure). AFT models describe treatment effects in which the occurrence of events is advanced (or delayed) in one group compared to the other by a constant proportion. For example, the time until first sexual intercourse may be delayed for participants exposed to a prevention program compared to control participants such that the probability that a treatment participant remains abstinent for any particular amount of time is the same as the probability that a control participant remains abstinent for only half as long. Although the requirement for the AFT model that event times follow a specific distribution is a more restrictive assumption that for the PH model, the AFT model encompasses a number of survival time distributions (e.g. generalized gamma, Weibull, log logistic, log normal) that accommodate many possible patterns of survival. In addition, because the AFT model uses time, rather than rate, in its dependent variable, it functions more similarly to a linear regression model than the PH model. Consequently, it may be advantageous to incorporate the AFT model into mediation analysis. Like the PH model, the AFT model can be estimated using standard statistical software, including SAS, the procedures of which will be highlighted in this paper.

The purpose of this paper is to familiarize readers with some of the main considerations involved in choosing an approach to conducting a mediation analysis when there are survival outcomes. These considerations are (1) how are the empirical parameter estimates from the procedures related to the casual effects in the model? (2) what are the statistical properties of the procedures? (3) what are the effects of censored outcomes? We compare the PH and AFT approaches for illustration. As in previous investigations of survival mediation (e.g., Tein and MacKinnon, 2001), the Weibull distribution will serve as the event time distribution and the comparison procedures will involve the SAS procedures PHREG and LIFEREG. The Weibull model (i.e., the model using the Weibull distribution) is the most appropriate for directly comparing the performance of PH and AFT procedures because no other model is both a PH model and an AFT model^1^. Furthermore, the Weibull model is versatile enough to potentially represent the range of distributions that are found in clinical research. The Weibull model can accommodate event times rates that are decreasing (e.g., situations where patients are most vulnerable to relapse immediately after a treatment ends with vulnerability decreasing thereafter), increasing (e.g., situations when the risk of relapse is minimal right after treatment, but the effects of treatment wear off over time and vulnerability increases over time), or constant.

In the present investigation, we will (1) compare mediation models and procedures that have often been applied to continuous outcomes to those involving PH and AFT models of continuous-time survival outcomes; (2) introduce the concepts of causally defined direct and indirect effects, and discuss the conditions under which survival mediation approaches using PHREG and LIFEREG allow these to be estimated; (3) report on results of simulations comparing statistical properties of mediation analyses involving PHREG and LIFEREG, under a variety of data conditions; (4) apply PHREG and LIFEREG approaches to a simulation dataset as an example, and; (5) discuss implications and recommendations. In the survival mediation models discussed, the independent variable will be a dichotomous treatment variable, the mediator a continuous variable measured at one or two time points, and the dependent variable a continuous-time survival variable following a Weibull distribution.

## Comparison of standard and survival mediation models

**Figure 1:**
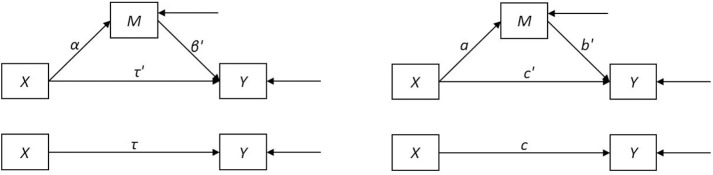
**Path diagrams for (partial) mediation model with unspecified error distributions and population coefficients (left) or sample coefficients (right)**. *X* represents the independent variable, *M* the mediator, and *Y* the outcome.

Figure 1 shows path diagrams for a general single-mediator model allowing for partial mediation, depicting the coefficient conventions that will be used in this paper.

In the three-variable partial mediation model for a continuous or dichotomous independent variable, a continuous mediator and continuous outcome, and normally-distributed errors, structural coefficient estimates are either simple or partial ordinary least squares (OLS) regression slopes. It is assumed that the independent variable and mediator are measured without error, and there is no independent variable-mediator interaction. We will refer to this model as the standard mediation model. In this case, the linear regression models corresponding to the path diagram are

(1) $$\begin{array}{rcl} Y & = & \gamma_{1}+\tau \prime X+\beta \prime M+\varepsilon_{1} \end{array}$$

(2) $$\begin{array}{rcl} M & = & \gamma_{2}+\alpha X+\varepsilon_{2} \end{array}$$

(3) $$\begin{array}{rcl} Y & = & \gamma_{3}+\tau X+\varepsilon_{3} \end{array}$$

where *Y* is the dependent variable, *X* is the independent variable, *M* is the mediator, each γ is the *Y*-intercept for the corresponding model, and each ε is an error term that follows a normal distribution with mean zero and is uncorrelated with the predictor variables in the corresponding model; ε_1_ and ε_2_ are assumed to be independent.

Let the population linear regression parameters α, β′, τ′, τ be estimated by sample values *a, b*′, *c*′, and *c*. Primed coefficients indicate relations between the same dependent and independent variables as corresponding unprimed coefficients, but adjusted for another variable, e.g., *c*′ is the relation between *X* and *Y*, adjusted for *M*, and *c* is the relation between *X* and *Y* without adjustment for another variable. Thus, the simple regression coefficient *c* reflects the estimated expected difference in *Y* for individuals one unit apart on *X*, whereas the partial regression coefficient *c*′ reflects the estimated expected difference in *Y* for individuals one unit apart on *X*, for constant *M*. If the model is true, *c* also reflects the estimated expected increase in *Y* for individuals increasing one unit on *X* (with the corresponding interpretations true for the other coefficients). Then, the total (causal) effect of *X* on *Y*, τ is estimated by *c*, the direct effect of *X* on *Y*, τ′, is estimated by *c*′, the indirect effect of *X* on *Y* through *M*, αβ′ is estimated by the product *ab*′, and the direct and indirect effects sum to the total effect. That is, τ = τ′ + αβ′ and *c* = *c*′ + *ab*′. Because of this equality, the difference *c* − *c*′ is equivalent to *ab*′, and is also an estimate of the indirect effect. These two methods of estimating the indirect effect are referred to as the difference of coefficients and product of coefficients methods, respectively (MacKinnon et al., 2002).

In a model with Weibull-distributed outcomes, the coefficients β′, τ′, τ may or may not be estimated by sample coefficients, depending on which survival analysis method is used. The SAS procedure LIFEREG allows one to assume a Weibull distribution for event time (T) and obtain estimates of these parameters for the log of the event time. In this case, the models corresponding to the path diagram are

(4) $$\begin{array}{rcl} Y & = & \log\left(T\right)=u_{1}+{\tau \prime X+\beta \prime M+\varepsilon }_{4} \end{array}$$

(5) $$\begin{array}{rcl} M & = & \gamma_{2}{+\alpha X+\varepsilon }_{2} \end{array}$$

(6) $$\begin{array}{rcl} Y & = & \log\left(T\right)=u_{3}{+\tau X+\varepsilon }_{6} \end{array}$$

where the dependent variable *Y* is the log of the event time, *u*_1_, γ_2_, and *u*_3_ are intercepts, and each ε is an error term. Taking the log of a Weibull distribution results in a two-parameter extreme value distribution. Thus, whereas ε_2_ follows a normal distribution, ε_4_ and ε_6_ follow a two-parameter extreme value distribution.

When the log transformation of time is the dependent variable, the equations look very much like those for the standard mediation model, and VanderWeele (2011a) has shown that the equality τ = τ′ + αβ′ holds true for the AFT model. The SAS procedure PHREG, on the other hand, does not estimate the coefficients β′, τ′, and τ, but rather β^*^′, τ^*^′, and τ^*^. Sample coefficients b^*^′, c^*^′, and c^*^ reflect estimated expected differences in log hazard rather than log survival time. Whereas a greater survival time indicates a longer time before an event occurs, a greater hazard rate indicates a shorter time before an event would occur. Therefore, coefficients produced by LIFEREG and PHREG procedures for the same data will be opposite in sign. The mediation-related models for the log hazard are

(7) $$\begin{array}{rcl} Y & = & \log h\left(T\right)=\eta_{1}\left(T\right)+\tau^{*\prime }X\;+\;\beta^{*\prime }M \end{array}$$

(8) $$\begin{array}{rcl} M & = & \gamma_{2}+\alpha X+\varepsilon_{2} \end{array}$$

(9) $$\begin{array}{rcl} Y & = & \log h\left(T\right)=\eta_{3}\left(T\right)\;+\;\tau^{*}X \end{array}$$

where *h*(*T*) is the hazard for the event at time *T*, and η(*T*) is the log of the hazard function for an individual with covariate values of 0 (i.e., the log of the baseline hazard function). Equations (7, 9) differ from the other equations presented in that there is no error term when the dependent variable is expressed in terms of the hazard rate; in the PH model, the same hazard rate is assumed to be shared by all participants sharing the same set of predictor values, and is associated with different survival times via stochastic processes.

## Causally defined direct and indirect effects

### Associational causal modeling

Mediation analysis as discussed by Baron and Kenny (1986) falls under the rubric of what we refer to as associational causal modeling (structural equation modeling falls under this rubric as well). Associational causal modeling arose from the work of Wright (1918, 1921, 1934), who noted that correlations between variables in path models without feedback loops could be “decomposed” into sums of standardized OLS regression coefficients, other correlation coefficients, and products of coefficients, according to a simple set of “tracing rules” applied to a path model. It is in this way that the total (causal) effect of *X* on *Y* in a three-variable mediation model estimated via OLS regression is decomposed into the sum of the direct and indirect (through *M*) effects using the equation *c* = *c*′ + *ab*′. It should be noted that regression coefficients only estimate causal effects if the causal model is true.

Assumptions of path models, including the standard mediation model, that are understood to allow for causal inferences include covariation, the correct temporal ordering of the variables and the lack of spurious covariation (Judd and Kenny, 1981). That is, it is accepted that there are three possible reasons that an independent and dependent variable covary: (1) the independent variable causes the dependent variable (as modeled), (2) the dependent variable causes the independent variable (reverse causation), or (3) both variables are caused by one or more “third” variables that are not included in the model (spurious causation). If the alternative explanations (2) and (3) are not true, then the first explanation is true by default. In a randomized treatment experiment, this logic allows one to accept treatment assignment as a cause of treatment differences because treatment assignment precedes outcome (ruling out reverse causation) and randomization makes spurious causation implausible. Thus, in a mediation model where *X* is the randomized treatment assignment, causal inferences can be made regarding the coefficients *a* and *c*, which estimate the causal weights of *X* on *M* and *X* on *Y*, respectively. However, because participants are not randomly assigned to levels of *M*, making causal inferences based on the covariation of *M* and *Y* is more problematic. It is generally accepted that results from a mediation analysis may suggest that a model is statistically plausible but they cannot prove that it is true. The plausibility of the model depends on the plausibility of the temporal ordering and the non-spuriousness assumptions.

A particular weakness of the associational causal modeling approach is that it is difficult to extend to different data types. For example, in forms of regression analysis in which the product of coefficients and the difference of coefficients method for estimating the indirect effect provide different answers (αβ′ does not equal τ − τ′, and therefore, *ab*′ does not equal *c* − *c*′), it is not immediately self-evident which, if either, is interpretable as an indirect effect. VanderWeele (2011a) has shown that for the AFT model, αβ′ always equals τ − τ′, but for the PH model, αβ^*^′ does not generally equal τ^*^ − τ^*^′.

### Potential outcomes causal modeling

More recently, researchers working with a form of causal modeling that arose from the work of Neyman (1923) and Fisher (1926), have taken an interest in mediation modeling (e.g., Robins and Greenland, 1992; Pearl, 2001; Robins, 2003). This form of causal modeling defines causal effects in terms of what are referred to as potential outcomes, and we thus refer to this endeavor as “potential outcomes causal modeling.” That is, for any comparison of two treatment conditions, the causal effect on an individual participant is the difference between the outcomes the participant would experience in the two treatment conditions (that is, the two potential outcomes for the participant). It is recognized that each participant is assigned to only one treatment, the potential outcome for which is observed. However, the potential outcome for the other treatment is not observed; it is referred to as the counterfactual outcome (Rubin, 1974; Holland, 1986). Because it is never the case that all of the potential outcomes are observed, it is also true that causal effects for individuals cannot be measured. It follows that average causal effects cannot be directly calculated and compared. However, it is sometimes possible to estimate average causal effects without bias using observed data. For example, in the case of a randomized experiment, the difference between the averages of observed outcomes for each treatment, calculated over the participants within each treatment, is an unbiased estimate of the average difference between potential treatment outcomes for each participant (Holland, 1986).

The inclusion of a mediator variable complicates the endeavor of defining potential outcomes and estimating causal effects. For each participant, each treatment condition is now associated with a potential value for the mediator variable as well as for the outcome variable. Additional potential values for the outcome variable under each treatment condition can be defined under the assumption that the mediator variable is set to the value associated with the other treatment condition. Then, for example, the direct effect of a treatment versus control condition can be defined as the difference between the potential values of the outcome variable for each treatment when the mediator variable is held at the potential value associated with the control treatment. This would reflect the effect of changing the condition from control to treatment without allowing the value of the mediator to change accordingly. Direct effects defined in this way, such that the value of the mediator is held constant within each individual but is allowed to vary among individuals, are referred to as natural direct effects. If it is imagined that the mediator can be set to values other than those associated with the treatment conditions, then potential values for the outcome variable could be defined for the each treatment condition at any possible value for the mediator. Direct effects defined at specific mediator levels to which it is imagined all individuals are set, are referred to as controlled direct effects.

Unlike the associational approach, the potential outcomes approach to causal modeling offers a clear, consistent definition for causal effects that can be applied regardless of data type, analysis procedure, or study design. It therefore can be used to assess the possible interpretations of proposed causal effect estimates. It has been shown that, if the temporal order and nonspurious^2^ associational causal modeling assumptions described above hold true, the direct, indirect, and total effect estimates obtained from OLS regression analyses are also estimates of natural causal effects according to the potential outcomes framework for the standard partial mediation model (Pearl, 2012). When analyzing data with non-typical outcomes via regression procedures (for example, logistic regression for binary outcomes, or proportional hazards regression for survival outcomes), it may be tempting to treat the resulting coefficients in the same manner as OLS regression coefficients to obtain estimates of direct, indirect, and total effects. However, it is not safe to assume that doing so will produce terms with causal interpretations. VanderWeele (2011a) has shown that, for the AFT model, not only does the equality τ = τ′ + αβ′ hold true, but the coefficients can represent natural causal effects in the log-survival time metric according to the potential outcomes framework, analogously to coefficients from OLS regression for typical continuous outcomes in the standard mediation model. VanderWeele (2011a) has also shown that, for the PH model, neither the equality of τ^*^ and the sum τ^*^′ + αβ^*^′ nor the interpretation of terms as causal effects hold true, except in the case of rare outcomes. Rare outcomes correspond to very high study duration censoring rates, which are not common in clinical research. When both the causal interpretations and the equality hold, there is an intuitive appeal to thinking about whether the mediated effect accounts for a large or small proportion of the total effect, using, for example, the proportion mediated ratio αβ′/τ′ + αβ′ estimated by *ab*′/(*c*′+*ab*′). Such ratios should be reported with caution, however, as they have been shown to be unstable except with very high sample sizes (MacKinnon et al., 1995).

## Simulation studies

We performed a series of simulation studies to examine statistical properties of mediation analyses involving PHREG and LIFEREG, under a variety of data conditions. Of particular interest was whether or not the finding of Tein and MacKinnon (2001) that, in a three-variable partial mediation model with Weibull distributed event times and without censoring, the equality *c* = *c*′ + *ab*′ was closely approximated by coefficients obtained using the LIFEREG procedure, would hold in the presence of censored event times. Not surprisingly, the equality c^*^ = c^*^′ + ab^*^′ was not approximated when using the PHREG procedure, even with no censoring. Also of interest was the ability of mediation analyses incorporating these procedures to estimate coefficients and their relative power and Type I error.

### Methods

To address these questions, a series of Monte Carlo studies was performed, with each simulated data set representing a randomized control treatment trial comparing two treatments with equal sample sizes. Each trial had an acute treatment phase, during which a putative mediator was assessed either once, or twice (to allow for an analysis where an initial score is covaried), and a follow-up phase, during which simulated participants were evaluated for the occurrence of the outcome event. Simulated data sets varied by: (1) inclusion of mediator intake score; (2) sample size; (3) mediation model; (4) mediation parameter values; and (5) censoring condition.

The standard deviation of each measurement of the mediator was set to equal that of the dichotomous treatment variable (0.5) so that differences in bias and power for the mediation coefficients would not reflect artifacts of differences in variability. Each trial had one of eight total sample sizes (20, 50, 100, 200, 500, 1000, 2000, or 5000), and one of eight mediation parameter combinations.

In addition to partial mediation, conditions of complete mediation and no mediation were modeled. The partial mediation parameters were set, following Tein and MacKinnon (2001), as follows: (α: 0.2, 0.4; β′: 0.2, 0.4, 0.6, 0.8; τ′: 0.2), with mean α of 0.3, mean β′ of 0.5, and mean indirect effect αβ′ of 0.15. For complete mediation, the parameters were: (α: 0.2, 0.4; β′: 0.2, 0.4, 0.6, 0.8; τ′: 0), with mean α of 0.3, mean β′ of 0.5, and mean indirect effect αβ′ of 0.15. For no mediation, eight parameter combinations were formed by setting τ′ to 0.2 and setting either α or β′ to 0 and varying the other parameter (0.2, 0.4, 0.6, 0.8); with mean α of 0.25, mean β′ of 0.25, and mean indirect effect αβ′ of 0.

Censoring was modeled in the following way. The outcome event may have been censored either because: (1) a participant was lost to follow-up (dropout) or (2) the follow-up period ended before the event occurred (study duration). Dropout censoring was modeled to be non-informative, which means that there was no relation between a participant's (observed or unobserved) event time and whether or not they were lost to follow-up. Data sets contained either no censoring, dropout censoring only, study duration censoring only, or both dropout and study duration censoring. Study duration and dropout censoring were modeled to follow distributions that were adjusted for each parameter combination to result in approximately 30% censoring of either form. When both study duration and dropout censoring were modeled together, roughly 40% of the outcome events were censored by the end of each simulated study. This range of censored observations should allow effects due to censoring to become apparent, and is not unreasonable to examine in the context of clinical research (Wierzbicki and Pekarik, 1993; Swift and Greenberg, 2012). Follow-up time was defined as the minimum of each individual's event time, dropout time, and study duration, as applicable, and served as the dependent variable.

Two sets of mediation analyses were performed on each data set. In one set of mediation analyses, we employed a combination of OLS regression (REG for paths, such as from treatment to mediator, that did not contain the survival outcome) and parametric AFT procedures regressing the log of survival time (LIFEREG for paths that contained the survival outcome). In the second set we also used REG for paths not containing the survival outcome and semiparametric PH procedures regressing the log hazard (PHREG) for paths containing the survival outcome. For each simulation, 500 replications were conducted. Results for point estimates of mediation parameters and power or Type I error were pooled over the eight parameter combinations for each sample size (i.e., 8 Í 500 = 4000 estimations) and used to compare mediation analysis procedures. Full details of the simulation can be found in Supplemental Materials. In the results reported below, LIFE/REG and PH/REG refer to mediation analysis procedures involving combinations of the REG procedures and the LIFEREG and PHREG procedures, respectively.

### Results

#### Censoring

Results from the current set of simulation studies suggest that the near equality of *c* = *c*′ + *ab*′ using LIFE/REG that was found in the absence of censoring (Tein and MacKinnon, 2001) fails to hold in the presence of censoring. This is especially true for censoring that occurs due to the limited duration of the follow-up period (study duration censoring). This inequality reflects bias in *c* such that the total effect of treatment on the outcome is underestimated. As a result, inferences made about mediation based on *c* − *c*′ in the presence of censoring would tend to underestimate the degree of mediation. Whereas *ab*′ (also *c*′) is relatively unaffected by censoring (especially at higher sample sizes), comparing *ab*′ to a value of *c* that is biased toward zero would lead to an overestimate of the degree to which the total effect is accounted for by the indirect effect. It is possible, using LIFE/REG, to obtain an alternative estimate for the purported total effect, which we designate *c2* (i.e., *c*′ + *ab*′), which is less subject to the biasing effects of censoring than *c*. In any one empirical context, it may not be clear how much bias would be expected in the value of *c*. When using LIFE/REG to conduct mediation analyses in the presence of censored outcomes, mediation inferences using both *c* and *c2* can be obtained and compared, and the results using *c2* can be emphasized if there are substantial differences. For PH/REG, censoring leads to bias in the corresponding coefficient, c^*^, but in this case the bias is such that the total effect of treatment on outcome is overestimated rather than underestimated. Mediation inferences can be reported with this in mind, but it is not possible to obtain an alternate estimate using the other coefficients.

#### 1-wave vs. 2-wave mediator measurement

Incorporating 2 waves of mediator measurement instead of one did not substantially change the pattern of results (a non-monotonic pattern was introduced into the relationship between sample size and Type I error rates for the LIFE/REG indirect effect in the no mediator case).

#### Mediation model

Results across the different mediation models were generally similar, though for complete and no mediation models, higher sample sizes are needed for PH/REG to estimate parameters with as little bias as for the partial mediation model. Because a LIFEREG parameter with a zero value will correspond to a PHREG parameter that also has a zero value, the PH/REG parameter estimates obtained when evaluating complete or no mediation models are somewhat more easily interpreted than estimates obtained for partial mediation models.

#### Power/Type I error

Under all conditions, LIFE/REG has superior power or Type I error compared to PH/REG in the detection of coefficients.

## An example using a simulation data set

**Figure 2:**
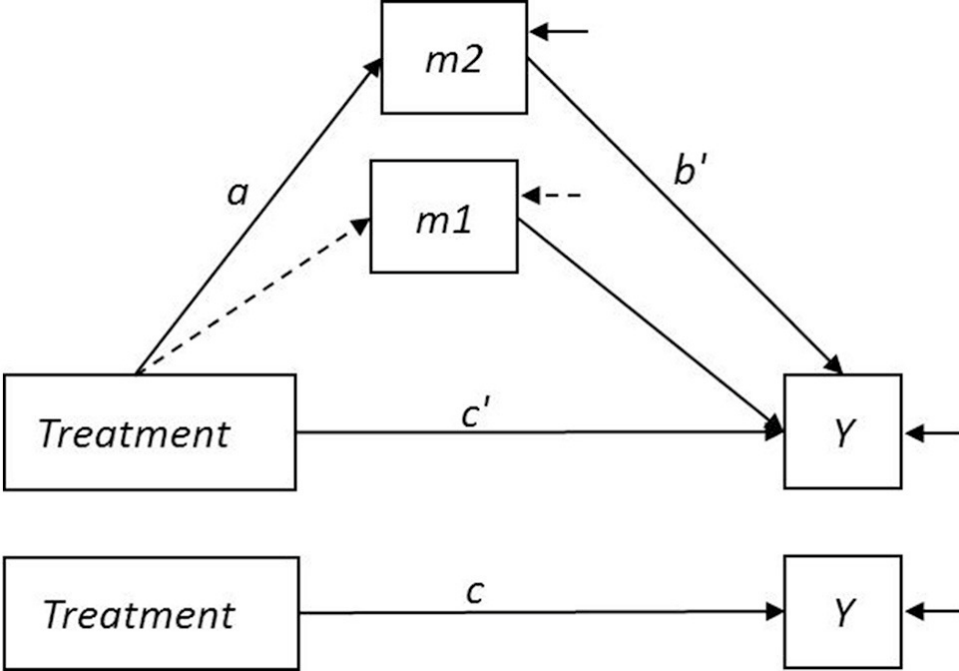
**Two-wave mediator model**. *Treatment* is a dichotomous variable. The variables *m1* and *m2* represent the mediator measured at intake and during treatment, respectively. The mediation-relevant paths are labeled according to the conventions shown in Figure 1. The dashed arrow between *Treatment* and *m1* indicates that in an RCT context, *Treatment* is assumed to be uncorrelated with the mediator at intake.

To illustrate the application of the mediation analysis approaches incorporating LIFEREG and PHREG, we chose a data set from the two-wave mediator study modeling partial mediation with a total sample size of 200 and α, β′, and τ′ parameters of 0.4, 0.4, and 0.2, respectively, and both types of censoring. The two wave mediator model corresponding to this simulation study is shown in Figure 2.

Imagine, for example, that the simulated data come from a study in which depressed outpatients recently remitted from depression after a course of antidepressant medication (ADM) are randomly assigned to one of two treatments for relapse prevention, such as ADM continuation alone or ADM continuation plus a brief course in mindfulness training. After this period, all patients continue on ADM and are periodically assessed for relapse for a fixed period of time. The variables *m1* and *m2* are measures of the mediator (for example, a measure of comfort with dysphoric sensations) taken at time 1 (intake interview) and time 2 (an interview during treatment), respectively. Although the mediator is measured at more than one time, it is not treated as a time-dependent covariate; both measurements occur before the period of monitoring for the event of interest. The variable *Y* represents the log of the time that passes between the end of the treatment period and the first relapse to depression. The time until relapse for a patient may be censored either because the patient was lost to follow-up (dropout censoring; in this example, for reasons assumed to be unrelated to their risk for relapse) or because when the follow-up period ended the patient had not experienced a relapse (study duration censoring). Because of random assignment to Treatment, the path from Treatment to *m1* is assumed to be zero, and the coefficient *c* can be estimated from the simple model predicting the outcome from treatment without controlling for *m1* or *m2*.

**Figure 3:**
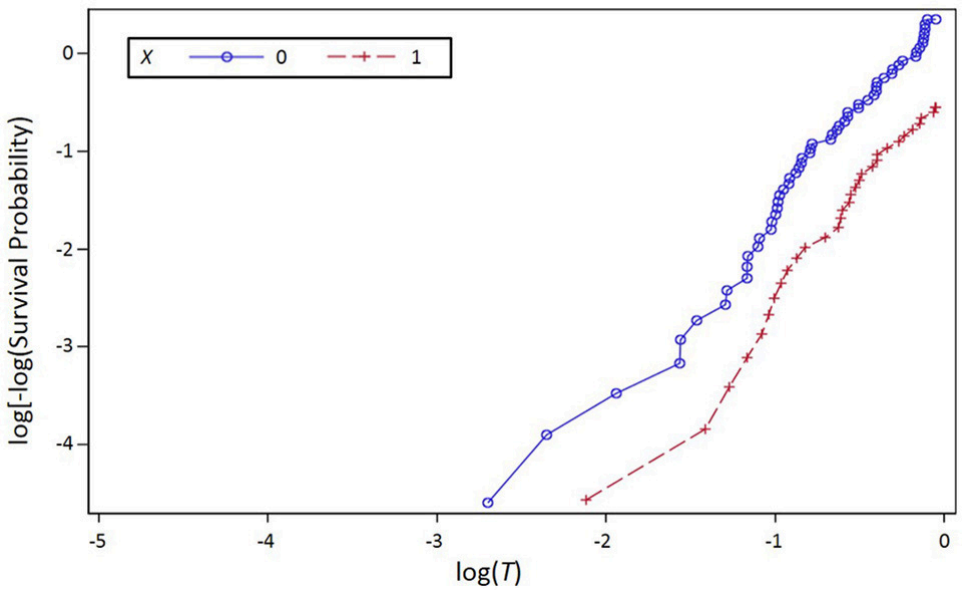
**Log of the negative log of the estimated survivor function plotted against the log of event time (*T*) for the simulation data set, stratified by Treatment (*X*), where *X* is a dichotomous variable with values 0 and 1 representing two treatments**.

A simple graphical method for simultaneously evaluating the assumptions that the event time for each treatment follows a Weibull distribution, and that the hazard functions for the two treatments are proportional, involves evaluating the log-log survivor functions for both treatments. A survivor function is the probability of surviving beyond any particular time; estimates of survivor functions are often depicted using Kaplan-Meier curves. The log of the negative log of the Weibull survivor function is a linear function of the log of time. Thus, empirical estimates of these values plotted against the log of time will produce a straight line for Weibull distributed data. The SAS syntax used to produce a plot for the simulated data set is:

PROC LIFETEST PLOTS = LLS; time t^*^censor(0); STRATA x; RUN;. The corresponding output is shown in Figure 3.

The reasonably straight lines in Figure 3 suggest that the Weibull assumption is not grossly violated. SAS syntax for the mediation analysis performed using the LIFEREG procedure and the Weibull assumption is found in the first section of Appendix A in Supplementary Materials. Output relevant to the mediation-related coefficients are in Table 1.

**Table 1:** **Relevant output from LIFE/REG mediation analysis of example data**.

**Parameter**	***D.F*.**	***Est*.**	***S.E*.**	***t*-value**	***p*-value**
**REG**					
*a*	1	0.50	0.74	6.74	<0.001
**Parameter**	***D.F***.	***Est***.	***S.E***.	**Chi-Square**	***p*****-value**
**LIFEREG1**					
*c*	1	0.41	0.11	14.6	<0.001
**LIFEREG2**					
*c*′	1	0.23	0.10	5.0	0.025
*b*′	1	0.40	0.09	21.7	<0.001

The results in Table 1 are consistent with a model of partial mediation. The total, direct, and indirect effects are expressed in terms of log survival time. For example, *c* (0.41) reflects the estimated expected difference in log time until relapse for the two treatment groups, which can be roughly decomposed into estimates of purported direct effects of treatment and indirect effects of treatment through self-correction of depressotypic belief of 0.23 and (0.50) (0.40) = 0.20, respectively. Because c has been shown to have a bias toward zero in the presence of censoring, one might use the value *c2* = *c*′ + *ab*′ (0.23 + [0.50][0.40] = 0.43) as an alternative estimate of the purported total effect. In either case, roughly half of the estimated total effect of treatment on log time until relapse is attributable to the estimated direct and indirect effects. The value of e^*c*2^ (e^.43^ = 1.54) provides the estimated ratio of mean survival times for the two treatment groups overall, and e*^c^*′ (e^0.23^ = 1.26) provides the same ratio for the two treatment groups, controlling for the mediator measured at intake and during treatment. (Note that a survival time ratio of 1 corresponds to no treatment difference). Thus, the expected time until relapse for the combined treatment is 54 percent greater than for ADM alone overall (compared to 50 percent using *c*), whereas it is only 26 percent greater when the mediator is controlled. The conclusion from this pattern of estimates is that combined treatment is more effective at delaying relapse than ADM alone, and this effect is less pronounced when the mediator is included in the model.

If the lines were not reasonably straight, suggesting that the Weibull assumption was grossly violated, one could use the plot in Figure 3 to evaluate the proportional hazards assumption. When two hazard functions are proportional, the log-logs of the corresponding respective survivor functions will differ by a constant; the lines in the plot will be parallel. The lines for the two treatments are (roughly) parallel, indicating no gross violation of the proportional hazards assumption; lines that are not straight but that are parallel would indicate violation of the Weibull assumption without violation of the proportional hazards assumption. Mediation analysis performed using PROC PHREG would use, in addition to the PROC REG code already introduced, the code found in Section 2 of Appendix A in Supplementary Materials. Relevant output from this analysis can be found in Table 2.

**Table 2:** **Relevant output from PH/REG mediation analysis of example data**.

**Parameter**	***D.F*.**	***Est*.**	***S.E*.**	**Chi-square**	***p-*****value**	
**REG**						
*a*	1	0.50	0.74	6.7	<0.001	
**Parameter**	***D.F***.	***Est***.	***S.E***.	**Chi-square**	***p-*****value**	**Hazard Ratio**
**PHREG1**						
c*	1	−0.87	0.22	15.0	<0.001	0.42
**PHREG2**						
c*′	1	−0.54	0.25	4.8	0.029	0.58
b*′	1	−0.94	0.20	21.9	<0.001	0.39

Using just the significance results from Table 2, the conclusion from the PH/REG analysis is similar to that found using LIFE/REG; the data are consistent with partial mediation. Sample coefficients b^*^′, c^*^′, and c^*^ reflect estimated expected differences in log hazard rather than log survival time, and their values cannot be used to estimate direct, indirect, or total effects. In accordance with the relationship between hazard and survival time, the coefficients obtained via PHREG are opposite in sign to those obtained via LIFEREG. The hazard ratio in the output is the exponentiation of the parameter estimate. The hazard ratio corresponding to e^c*^(e^−0.87^ = 0.42) provides the estimated hazard ratio for the two treatment groups overall, and the hazard ratio corresponding to e^c^*′ (e^−0.54^ = 0.58) provides the same ratio for the two treatment groups, controlling for the two measures of the mediator. Thus, the hazard of relapse for participants in combined treatment is estimated to be 42 percent of the hazard of relapse for participants in ADM alone, overall, while it is estimated to be 58% when the mediator is controlled; combined treatment is more effective at reducing the hazard of relapse (which corresponds to delaying relapse) than ADM alone, and this effect is less pronounced when the mediator is controlled. (Note that a hazard ratio of 1 would correspond to no treatment difference). Although c^*^ may have a bias away from zero due to the presence of censored data (and the hazard ratio may consequently be biased away from 1), an alternate estimate cannot be obtained from combining c^*^′ and ab^*^′.

## Discussion

The purpose of this paper was to introduce several considerations to take into account when choosing a method for performing a mediation analysis with survival outcomes. The PH model is commonly used to assess survival data and the AFT model is an alternative model that, like the PH model, has had limited evaluation in the context of mediation analysis. Although a comparison of procedures for these models as applied to Weibull distributed event times was used for illustration, the issues that arose would be relevant when considering approaches not included in this paper.

One of the issues that arises in mediation analysis with survival outcomes (and other nonstandard models) is that analytic procedures may not produce coefficients that can be interpreted as estimates of the indirect effect of treatment on outcome using either the product of coefficients or the difference of coefficients methods. They also may not provide valid estimates of the direct effect of treatment on outcome. The potential outcomes framework allows the determination of appropriate casual estimates regardless of data type. One advantage of the LIFE/REG procedure for estimating AFT models over the PH/REG procedure for estimating PH models is that, in the absence of interactions and other nonlinear effects, the LIFE/REG procedure always produces coefficients that can provide valid causal estimates, while the PH/REG procedure only does so in the context of rare outcomes (VanderWeele, 2011a). In addition, the indirect and direct effects from the LIFE/REG procedure sum to the total effects in the log time metric, allowing for intuitive estimates of the size of the indirect effect.

A second issue to consider involves the statistical properties of the methods under consideration. Regarding both power and Type I error, the LIFE/REG procedure is superior to the PH/REG procedure. This is true under a number of data conditions (2-waves of mediator, different types of mediation, and different types of censoring). In terms of estimating parameters, both procedures are less robust to study duration censoring than they are to dropout censoring. Censored event times, especially those due to study duration, affected the *c* and c^*^ coefficients more than the others. The *c* and c^*^ coefficients were biased in opposite directions such that in LIFE/REG the total effect of *X* on *Y*, without regard to *M*, is underestimated and in PH/REG this effect is overestimated. When using LIFE/REG, this bias can be addressed by using *c*′ + *ab*′ as an alternative estimate of *c*, as illustrated in the simulation example. There is no comparable method for addressing the overestimation bias in PH/REG.

One implication of these findings is that it could be useful for researchers interested in performing mediation analyses with survival data to check for violations of AFT assumptions rather than resorting to PH modeling as the default. If Weibull assumptions are not grossly violated, LIFE/REG is to be preferred over PH/REG in mediation analyses. When the Weibull assumption is violated but the proportional hazards assumption is not, PH/REG can be applied, although direct and indirect effect estimates are likely inaccurate (except for rare outcomes), and results obtained in the presence of censored outcomes should be reported with caution, due to the overestimation bias for the total effect. When both Weibull and proportional hazards assumptions are violated, it is possible that event times follow an alternative AFT distribution, and can be subjected to mediation analysis via LIFE/REG. We did not investigate methods for performing mediation analysis involving survival data that are neither PH nor AFT. Incorporating methods for analyzing such data into mediation analysis is a topic worthy of further investigation. We also did not investigate methods for performing mediation analysis on models that incorporate time-dependent mediators, non-continuous mediators, treatment by mediator interactions, or informative censoring. One advantage to PH methods is that, unlike fully parametric models, they allow for the inclusion of time-dependent covariates, that is, predictors that may change in value during the follow-up period for event times; these theoretically include time-dependent mediators. However, the estimation and interpretation of causal effects in the context of predictors, while sometimes possible, is more complicated than the application of methods covered in this paper (e.g., Robins and Hernan, 2009). The extension of the methods discussed here to models involving non-continuous mediators and treatment x mediator interactions is more straightforward, and a macro is available to assist with analyses (Valeri and VanderWeele, 2015). The assumption of non-informative censoring is violated when loss to follow-up is related to event time, such as when patients who are feeling more depressed and are more likely to relapse soon are also more likely to avoid follow-up interviews. Such violations may occur in many clinical research contexts; incorporating the modeling of informative censoring into mediation analysis would be a particularly useful focus of future investigations.

It is important keep in mind that, regardless of mediation analysis method applied, the ability of regression coefficients to estimate causal effects is conditional upon the correct temporal ordering of variables and the lack of spuriousness in the covariation of *X* and *Y*, and *M* and *Y*. The temporal ordering of variables can, at times, be at least partially established via study design. Although the plausibility of the lack of spuriousness between *M* and *Y* is often in question, sensitivity analyses can be used to assess the effects of violations of this assumption (e.g., Imai et al., 2010b; VanderWeele, 2011b; Cox et al., 2013; MacKinnon and Pirlott, 2015).

## Author contributions

LG, DM, and RD contributed to conception and design of the work. LG and AB contributed to analyses and interpretation of data for the work. LG drafted the work. All four authors made substantial contributions to revising the work. All gave final approval for the submission and agree to be accountable for all aspects of the work.

## Funding

This work was supported by NIMH grant 3-R01-MH060998-09S1.

### Conflict of interest statement

The authors declare that the research was conducted in the absence of any commercial or financial relationships that could be construed as a potential conflict of interest.

## Footnotes

^1^The exponential model is also both PH and AFT, but it is nested within and can be considered a version of the Weibull model.

^2^The nonspuriousness assumptions in the potential outcome literature are actually somewhat more elaborate, and are typically outlined in the four following necessary, but not sufficient, conditions (Pearl, 2001; VanderWeele, 2010): (1) No unmeasured confounders of the relation between *X* and *Y*; (2) No unmeasured confounders of the relation between *M* and *Y*; (3) No unmeasured confounders of the relation between *X* and *M*; (4) No measured or unmeasured confounders of *M* and *Y* that have been affected by *X*. Assumptions (1) and (3) refer to the ignorability of treatment assignment (i.e., treatment assignment is independent of potential outcomes for the mediator and outcome variables) given observed pretreatment confounders. These assumptions are satisfied with randomized treatment assignment. Assumption (2) refers to the ignorability of mediator assignment given the observed treatment assignment and pretreatment confounders. (i.e., mediator assignment is independent of potential outcomes for the outcome variable). The ignorability of the mediator assumption is difficult to meet because assignment of *M* is not externally controlled in most studies (e.g., the mediator status is not randomly assigned, but is rather self-selected by participants). Even when conditioning on observed confounders for the relation between *M* and *Y*, there can still be unobserved confounders that would lead to violation of this assumption. This assumption is strong and is usually ignored in studies even though a causal interpretation of a mediated effect is not possible without satisfying this assumption (MacKinnon, 2008; VanderWeele and Vansteelandt, 2009). Assumption (4) can be even more difficult to satisfy, because it is not possible to mitigate the impact of this type of confounder by measuring and conditioning on it. Methods described in this article make assumptions (3) and (4) but approaches to investigate and adjust for violations of these assumptions are being developed (Imai et al., 2010a,b; VanderWeele, 2010; Coffman and Zhong, 2012; Cox et al., 2013).
