# Supplementary material for: Mediation Analysis with Survival Outcomes: Accelerated Failure Time vs. Proportional Hazards Models
Source: Front Psychol. 2016 Mar 30;7:423. doi: 10.3389/fpsyg.2016.00423 (PMC4811962; doi:10.3389/fpsyg.2016.00423)
Supplement: Supplementary file 1 [file Table1.pdf]

Appendix A

Example SAS Syntax

```
/*SECTION 1: Generating Table 1 using LIFE/REG*/
/*REG*/
proc reg; model m1 = x; model m2 = x m1; run;
/*LIFEREG1*/
proc lifereg; model t*censor(0) = x; run;
/*LIFEREG2*/
proc lifereg; model t*censor(0) = x m2 m1; run;
/*SECTION 2: Generating Table 2 using PH/REG*/
/*PHREG1*/
proc phreg; model t*censor(0) = x; run;
/*PHREG2*/
proc phreg; model t*censor(0) = x m2 m1; run;
```

Appendix B

Supplemental Material: Detailed Simulation Results

Table B1

Mean values for parameter estimates for 1-wave mediator (partial mediation) using LIFEREG

Method									
Sample Size		20	50	100	200	500	1000	2000	5000
REG	*a*	.298	.301	.299	.302	.301	.300	.300	.300
No censoring									
LIFEREG	*b*'	.496	.501	.499	.501	.500	.500	.500	.500
	c'	.205	.199	.202	.199	.201	.200	.200	.200
	c	.354	.352	.352	.351	.351	.350	.350	.351
LIFE/REG	*ab*'	.148	.151	.149	.151	.150	.150	.150	.150
	c-*c*'	.150	.153	.150	.152	.150	.150	.150	.150
	ab'-(*c*-*c*')	-.001	-.002	.000	.000	.000	.000	.000	.000
Dropout censoring									
LIFEREG	*b*'	.508	.506	.499	.503	.501	.500	.500	.500
	c'	.202	.196	.202	.198	.200	.200	.201	.200
	c	.352	.345	.348	.347	.347	.346	.347	.347
LIFE/REG	*ab*'	.152	.152	.149	.152	.150	.150	.150	.150
	c-*c*'	.150	.149	.146	.149	.147	.146	.146	.146
	ab'-(*c*-*c*')	.002	.004	.003	.003	.004	.004	.004	.004
Study duration censoring									
LIFEREG	*b*'	.537	.517	.505	.505	.502	.501	.500	.500
	c'	.208	.198	.203	.197	.201	.200	.200	.201
	c	.351	.336	.335	.332	.332	.331	.331	.332
LIFE/REG	*ab*'	.160	.155	.151	.152	.151	.150	.150	.150
	c-*c*'	.143	.137	.132	.135	.132	.131	.131	.131
	ab'-(*c*-*c*')	.017	.018	.019	.018	.019	.019	.019	.019
Both dropout and study duration censoring									
LIFEREG	*b*'	.542	.521	.506	.506	.502	.501	.500	.500
	c'	.225	.198	.203	.197	.200	.201	.201	.201
	c	.376	.338	.338	.335	.334	.334	.334	.334
LIFE/REG	*ab*'	.161	.157	.151	.153	.151	.150	.150	.150
	c-*c*'	.151	.140	.135	.137	.134	.133	.133	.133
	ab'-(*c*-*c*')	.010	.017	.016	.015	.017	.017	.017	.017

Note. Mean parameter values: *a*=.30, *b*'=.50, *c*'=.20, *c*=.35, *ab*'=.15, *c*-*c*'=.15, *ab*'-(*c*-*c*')=0.

Table B2

Mean values for parameter estimates for 1-wave mediator (partial mediation) using PHREG

Method									
Sample Size		20	50	100	200	500	1000	2000	5000
No censoring									
	*b**'	-1.390	-1.307	-1.274	-1.266	-1.253	-1.252	-1.251	-1.250
PHREG	*c**'	-.582	-.518	-.516	-.502	-.503	-.501	-.502	-.501
	*c**	-.772	-.704	-.693	-.680	-.677	-.673	-.673	-.673
	*ab**'	-.414	-.393	-.381	-.382	-.377	-.376	-.375	-.376
PH/REG	*c**-*c**'	-.189	-.186	-.176	-.178	-.173	-.172	-.171	-.172
	*ab**'-(*c**-*c**')	-.224	-.206	-.205	-.204	-.203	-.203	-.204	-.204
Dropout censoring									
	*b**'	-1.469	-1.329	-1.282	-1.270	-1.256	-1.253	-1.251	-1.251
PHREG	*c**'	-.649	-.512	-.517	-.501	-.502	-.501	-.502	-.501
	*c**	-.865	-.725	-.719	-.705	-.701	-.698	-.698	-.698
	*ab**'	-.439	-.400	-.384	-.383	-.377	-.376	-.375	-.376
PH/REG	*c**-*c**'	-.217	-.213	-.202	-.205	-.199	-.196	-.196	-.196
	*ab**'-(*c**-*c**')	-.222	-.188	-.182	-.179	-.179	-.180	-.179	-.180
Study duration censoring									
	*b**'	-1.441	-1.324	-1.277	-1.270	-1.256	-1.253	-1.251	-1.251
PHREG	*c**'	-.565	-.506	-.512	-.496	-.502	-.500	-.501	-.501
	*c**	-.781	-.732	-.731	-.722	-.722	-.721	-.721	-.722
	*ab**'	-.429	-.397	-.382	-.383	-.377	-.376	-.375	-.376
PH/REG	*c**-*c**'	-.216	-.225	-.219	-.226	-.220	-.220	-.220	-.221
	*ab**'-(*c**-*c**')	-.213	-.172	-.164	-.157	-.157	-.156	-.155	-.155
Both dropout and study duration censoring									
	*b**'	-1.503	-1.341	-1.285	-1.273	-1.257	-1.254	-1.252	-1.251
PHREG	*c**'	-.662	-.506	-.515	-.496	-.501	-.502	-.502	-.501
	*c**	-.894	-.743	-.744	-.733	-.731	-.731	-.731	-.731
	*ab**'	-.451	-.403	-.384	-.384	-.378	-.376	-.375	-.376
PH/REG	*c**-*c**'	-.231	-.236	-.230	-.237	-.230	-.230	-.229	-.230
	*ab**'-(*c**-*c**')	-.220	-.167	-.154	-.148	-.148	-.147	-.146	-.146

Table B3

Power to detect non-zero effects for 1-wave mediator (partial mediation) using LIFEREG

Method									
Sample Size		20	50	100	200	500	1000	2000	5000
REG	*a*	.262	.529	.741	.910	.997	1.000	1.000	1.000
No censoring									
LIFEREG	*b*'	.648	.835	.918	.982	1.000	1.000	1.000	1.000
	c'	.257	.407	.682	.917	.999	1.000	1.000	1.000
	c	.437	.688	.909	.991	1.000	1.000	1.000	1.000
LIFE/REG	*a* & *b*'	.167	.445	.681	.892	.997	1.000	1.000	1.000
	mediation	.115	.379	.653	.888	.996	1.000	1.000	1.000
Dropout censoring									
LIFEREG	*b*'	.572	.787	.878	.956	.999	1.000	1.000	1.000
	c'	.214	.304	.527	.799	.992	1.000	1.000	1.000
	c	.345	.576	.825	.965	1.000	1.000	1.000	1.000
LIFE/REG	*a* & *b*'	.147	.418	.652	.868	.996	1.000	1.000	1.000
	mediation	.082	.307	.584	.846	.996	1.000	1.000	1.000
Study duration censoring									
LIFEREG	*b*'	.567	.791	.876	.962	1.000	1.000	1.000	1.000
	c'	.170	.300	.539	.802	.994	1.000	1.000	1.000
	c	.276	.566	.835	.974	1.000	1.000	1.000	1.000
LIFE/REG	*a* & *b*'	.145	.418	.651	.875	.996	1.000	1.000	1.000
	mediation	.073	.310	.594	.861	.996	1.000	1.000	1.000
Both dropout and study duration censoring									
LIFEREG	*b*'	.506	.742	.843	.940	.998	1.000	1.000	1.000
	c'	.155	.239	.432	.705	.979	1.000	1.000	1.000
	c	.224	.474	.750	.943	1.000	1.000	1.000	1.000
LIFE/REG	*a* & *b*'	.125	.393	.627	.853	.994	1.000	1.000	1.000
	mediation	.049	.250	.531	.821	.994	1.000	1.000	1.000

Table B4

Power to detect non-zero effects for 1-wave mediator (partial mediation) using PHREG

Method									
Sample Size		20	50	100	200	500	1000	2000	5000
No censoring									
PHREG	*b**'	.572	.818	.910	.981	1.000	1.000	1.000	1.000
	*c**'	.169	.355	.649	.904	.999	1.000	1.000	1.000
	*c**	.304	.625	.888	.988	1.000	1.000	1.000	1.000
PH/REG	*a* & *b**'	.145	.435	.676	.891	.997	1.000	1.000	1.000
	mediation	.078	.342	.626	.848	.955	.981	.995	1.000
Dropout censoring									
PHREG	*b**'	.465	.757	.866	.954	.999	1.000	1.000	1.000
	*c**'	.108	.258	.487	.779	.991	1.000	1.000	1.000
	*c**	.206	.503	.787	.959	1.000	1.000	1.000	1.000
PH/REG	*a* & *b**'	.114	.402	.642	.866	.996	1.000	1.000	1.000
	mediation	.042	.257	.545	.819	.969	.993	.999	1.000
Study duration censoring									
PHREG	*b**'	.519	.784	.875	.961	1.000	1.000	1.000	1.000
	*c**'	.135	.283	.531	.798	.995	1.000	1.000	1.000
	*c**	.251	.550	.828	.973	1.000	1.000	1.000	1.000
PH/REG	*a* & *b**'	.132	.416	.650	.873	.996	1.000	1.000	1.000
	mediation	.063	.299	.586	.854	.988	.999	1.000	1.000
Both dropout and study duration censoring									
PHREG	*b**'	.433	.732	.837	.937	.998	1.000	1.000	1.000
	*c**'	.098	.226	.420	.696	.980	1.000	1.000	1.000
	*c**	.185	.457	.740	.941	1.000	1.000	1.000	1.000
PH/REG	*a* & *b**'	.106	.386	.621	.851	.994	1.000	1.000	1.000
	mediation	.035	.236	.517	.810	.987	.999	1.000	1.000

Table B5

Mean values for parameter estimates for 1-wave mediator (complete mediation) using LIFEREG

Method									
Sample Size		20	50	100	200	500	1000	2000	5000
REG	*a*	.298	.301	.299	.303	.300	.299	.300	.300
No censoring									
	b'	.494	.501	.499	.501	.500	.500	.500	.500
LIFEREG	*c*'	.004	-.001	.002	-.001	.001	.000	.001	.000
	c	.153	.152	.152	.150	.151	.149	.150	.150
	ab'	.148	.151	.149	.151	.150	.150	.150	.150
LIFE/REG	*c*-*c*'	.149	.153	.150	.151	.150	.150	.150	.150
	ab'-(*c*-*c*')	-.001	-.002	.000	.000	.000	.000	.000	.000
Dropout censoring									
	b'	.504	.506	.499	.502	.500	.500	.500	.500
LIFEREG	*c*'	.004	-.004	.001	-.002	.000	.000	.001	.000
	c	.155	.148	.149	.149	.148	.148	.149	.149
	ab'	.151	.152	.149	.152	.150	.150	.150	.150
LIFE/REG	*c*-*c*'	.151	.151	.148	.151	.148	.148	.148	.148
	ab'-(*c*-*c*')	.000	.001	.001	.001	.002	.002	.002	.002
Study duration censoring									
	b'	.534	.518	.504	.505	.502	.501	.500	.500
LIFEREG	*c*'	-.002	-.004	.003	-.003	.000	.000	.001	.000
	c	.148	.143	.143	.141	.141	.140	.140	.141
	ab'	.159	.156	.151	.153	.151	.150	.150	.150
LIFE/REG	*c*-*c*'	.150	.147	.140	.144	.140	.140	.140	.140
	ab'-(*c*-*c*')	.010	.009	.010	.009	.010	.010	.010	.010
Both dropout and study duration censoring									
	b'	.536	.521	.504	.506	.502	.501	.500	.500
LIFEREG	*c*'	.006	-.006	.002	-.003	.000	.000	.001	.000
	c	.159	.142	.144	.143	.142	.141	.142	.142
	ab'	.160	.157	.151	.153	.151	.150	.150	.150
LIFE/REG	*c*-*c*'	.153	.148	.142	.145	.142	.141	.141	.141
	ab'-(*c*-*c*')	.007	.009	.008	.007	.009	.009	.009	.009

Note. Mean parameter values: *a*=.30, *b*'=.50, *c*'=0, *c*=.15, *ab*'=.15, *c*-*c*'=.15, *ab*'-(*c*-*c*')=0.

Table B6

Mean values for parameter estimates for 1-wave mediator (complete mediation) using PHREG

Method									
Sample Size		20	50	100	200	500	1000	2000	5000
No censoring									
	*b**'	-1.388	-1.308	-1.273	-1.265	-1.254	-1.252	-1.251	-1.250
PHREG	*c**'	-.012	.004	-.005	.002	-.001	.001	-.001	-.001
	*c**	-.318	-.290	-.285	-.280	-.278	-.275	-.276	-.276
	*ab**'	-.415	-.393	-.381	-.382	-.376	-.376	-.375	-.376
PH/REG	*c**-*c**'	-.306	-.295	-.280	-.282	-.276	-.276	-.275	-.275
	*ab**'-(*c**-*c**')	-.109	-.098	-.101	-.101	-.100	-.100	-.100	-.101
Dropout censoring									
	*b**'	-1.465	-1.333	-1.280	-1.269	-1.256	-1.253	-1.251	-1.251
PHREG	*c**'	-.021	.013	-.003	.005	.000	.000	-.002	-.001
	*c**	-.353	-.298	-.297	-.292	-.289	-.287	-.289	-.288
	*ab**'	-.439	-.402	-.383	-.384	-.377	-.376	-.375	-.376
PH/REG	*c**-*c**'	-.332	-.311	-.294	-.296	-.289	-.288	-.287	-.287
	*ab**'-(*c**-*c**')	-.107	-.091	-.089	-.087	-.088	-.089	-.088	-.089
Study duration censoring									
	*b**'	-1.433	-1.325	-1.273	-1.268	-1.256	-1.253	-1.251	-1.250
PHREG	*c**'	.003	.011	-.007	.006	-.001	.001	-.001	-.001
	*c**	-.323	-.306	-.308	-.301	-.301	-.299	-.300	-.301
	*ab**'	-.428	-.398	-.381	-.383	-.377	-.376	-.375	-.376
PH/REG	*c**-*c**'	-.325	-.317	-.301	-.307	-.300	-.300	-.299	-.300
	*ab**'-(*c**-*c**')	-.103	-.081	-.080	-.076	-.077	-.076	-.076	-.076
Both dropout and study duration censoring									
	*b**'	-1.497	-1.345	-1.279	-1.272	-1.256	-1.254	-1.251	-1.251
PHREG	*c**'	-.027	.016	-.005	.007	.000	.000	-.002	-.001
	*c**	-.367	-.308	-.313	-.307	-.305	-.304	-.305	-.305
	*ab**'	-.448	-.404	-.382	-.384	-.377	-.376	-.375	-.376
PH/REG	*c**-*c**'	-.340	-.324	-.308	-.314	-.305	-.304	-.304	-.304
	*ab**'-(*c**-*c**')	-.108	-.081	-.075	-.071	-.073	-.072	-.071	-.071

Table B7

Power to detect non-zero effects for 1-wave mediator (complete mediation) using LIFEREG

Method									
Sample Size		20	50	100	200	500	1000	2000	5000
REG	*a*	.262	.534	.742	.912	.997	1.000	1.000	1.000
No censoring									
LIFEREG	*b*'	.647	.834	.917	.983	1.000	1.000	.647	.834
	c'	.096	.072	.059	.053	.054	.055	.052	.046
	c	.188	.253	.360	.520	.730	.855	.943	.990
LIFE/REG	*a* & *b*'	.167	.447	.682	.896	.997	1.000	1.000	1.000
	mediation	.069	.182	.318	.504	.728	.854	.942	.989
Dropout censoring									
LIFEREG	*b*'	.574	.788	.882	.961	.999	1.000	.574	.788
	c'	.106	.070	.058	.052	.053	.057	.050	.044
	c	.158	.203	.295	.444	.658	.807	.915	.978
LIFE/REG	*a* & *b*'	.149	.421	.655	.875	.996	1.000	1.000	1.000
	mediation	.044	.133	.254	.430	.657	.806	.915	.978
Study duration censoring									
LIFEREG	*b*'	.555	.787	.875	.961	1.000	1.000	.555	.787
	c'	.068	.056	.050	.050	.050	.054	.050	.052
	c	.099	.170	.287	.441	.665	.817	.919	.981
LIFE/REG	*a* & *b*'	.143	.419	.650	.875	.997	1.000	1.000	1.000
	mediation	.034	.127	.257	.428	.663	.816	.918	.981
Both dropout and study duration censoring									
LIFEREG	*b*'	.497	.741	.846	.938	.998	1.000	.497	.741
	c'	.079	.060	.051	.053	.046	.055	.052	.049
	c	.095	.144	.249	.401	.603	.769	.888	.970
LIFE/REG	*a* & *b*'	.129	.393	.629	.853	.995	1.000	1.000	1.000
	mediation	.013	.090	.209	.374	.598	.764	.887	.968

Table B8

Power to detect non-zero effects for 1-wave mediator (complete mediation) using PHREG

Method									
Sample Size		20	50	100	200	500	1000	2000	5000
No censoring									
PHREG	*b**'	.570	.817	.910	.982	1.000	1.000	.570	.817
	*c**'	.057	.062	.057	.053	.054	.055	.051	.046
	*c**	.107	.192	.309	.487	.706	.850	.939	.989
PH/REG	*a* & *b**'	.145	.437	.676	.895	.997	1.000	1.000	1.000
	mediation	.037	.142	.278	.474	.704	.847	.938	.989
Dropout censoring									
PHREG	*b**'	.464	.762	.872	.959	.999	1.000	.464	.762
	*c**'	.058	.056	.055	.052	.051	.058	.050	.043
	*c**	.084	.153	.256	.415	.640	.801	.913	.978
PH/REG	*a* & *b**'	.118	.404	.648	.874	.996	1.000	1.000	1.000
	mediation	.019	.102	.224	.401	.637	.800	.912	.978
Study duration censoring									
PHREG	*b**'	.516	.781	.873	.961	1.000	1.000	.516	.781
	*c**'	.049	.054	.051	.050	.049	.053	.051	.052
	*c**	.090	.162	.276	.435	.661	.816	.918	.981
PH/REG	*a* & *b**'	.133	.417	.650	.875	.996	1.000	1.000	1.000
	mediation	.029	.123	.246	.420	.660	.814	.917	.980
Both dropout and study duration censoring									
PHREG	*b**'	.433	.726	.842	.935	.998	1.000	.433	.726
	*c**'	.050	.054	.050	.052	.045	.055	.051	.050
	*c**	.072	.137	.240	.390	.600	.766	.889	.969
PH/REG	*a* & *b**'	.109	.385	.626	.852	.995	1.000	1.000	1.000
	mediation	.013	.090	.209	.374	.598	.764	.887	.968

Table B9

Mean values for parameter estimates for 1-wave mediator (no mediation) using LIFEREG

Method									
Sample Size		20	50	100	200	500	1000	2000	5000
REG	*a*	.248	.251	.249	.252	.251	.249	.250	.250
No censoring									
LIFEREG	*b*'	.243	.251	.249	.251	.250	.250	.250	.250
	c'	.205	.199	.203	.199	.201	.200	.201	.200
	c	.202	.201	.202	.200	.201	.200	.201	.200
LIFE/REG	*ab*'	-.002	.000	-.001	.001	.000	.000	.000	.000
	c-*c*'	-.003	.002	.000	.001	.000	.000	.000	.000
	ab'-(*c*-*c*')	.001	-.002	.000	.000	.000	.000	.000	.000
Dropout censoring									
LIFEREG	*b*'	.250	.253	.249	.252	.250	.250	.250	.250
	c'	.207	.197	.202	.199	.200	.200	.201	.200
	c	.204	.198	.201	.200	.199	.199	.200	.200
LIFE/REG	*ab*'	-.001	.001	-.001	.001	.000	.000	.000	.000
	c-*c*'	-.002	.001	-.002	.001	-.001	-.001	-.001	-.001
	ab'-(*c*-*c*')	.001	.000	.001	.001	.001	.001	.001	.001
Study duration censoring									
LIFEREG	*b*'	.264	.260	.252	.253	.251	.250	.250	.250
	c'	.217	.201	.204	.199	.201	.200	.201	.200
	c	.207	.196	.198	.195	.195	.195	.195	.195
LIFE/REG	*ab*'	-.001	.000	.000	.001	.000	.000	.000	.000
	c-*c*'	-.010	-.005	-.006	-.004	-.006	-.005	-.005	-.005
	ab'-(*c*-*c*')	.008	.006	.006	.005	.006	.005	.005	.005
Both dropout and study duration censoring									
LIFEREG	*b*'	.267	.262	.253	.254	.252	.251	.250	.250
	c'	.229	.201	.204	.199	.200	.201	.201	.200
	c	.223	.196	.198	.196	.195	.196	.196	.196
LIFE/REG	*ab*'	-.001	.001	-.001	.001	.000	.000	.000	.000
	c-*c*'	-.006	-.005	-.006	-.003	-.005	-.005	-.005	-.005
	ab'-(*c*-*c*')	.005	.005	.005	.004	.005	.005	.005	.005

Note. Mean parameter values: *a*=.25, *b*'=.25, *c*'=.20, *c*=.20, *ab*'=0, *c*-*c*'=0, *ab*'-(*c*-*c*')=0.

Table B10

Mean values for parameter estimates for 1-wave mediator (no mediation) using PHREG

Method									
Sample Size		20	50	100	200	500	1000	2000	5000
No censoring									
PHREG	*b*'*	-.691	-.653	-.634	-.635	-.626	-.625	-.625	-.625
	c'*	-.574	-.519	-.519	-.504	-.503	-.500	-.502	-.501
	*c**	-.495	-.461	-.460	-.450	-.449	-.447	-.448	-.447
PH/REG	*ab*'*	.002	.000	.001	-.003	.001	.000	.000	.000
	c-c*'*	.079	.058	.059	.053	.055	.053	.054	.054
	ab'-(c*-c*')*	-.077	-.058	-.058	-.056	-.054	-.053	-.054	-.054
Dropout censoring									
PHREG	*b*'*	-.725	-.665	-.637	-.636	-.628	-.627	-.625	-.626
	c'*	-.611	-.518	-.519	-.504	-.503	-.501	-.503	-.501
	*c**	-.530	-.466	-.465	-.458	-.454	-.454	-.456	-.454
PH/REG	*ab*'*	.004	-.002	.002	-.003	.001	-.001	.000	.000
	c-c*'*	.082	.051	.053	.046	.048	.047	.047	.047
	ab'-(c*-c*')*	-.078	-.053	-.052	-.049	-.048	-.047	-.047	-.047
Study duration censoring									
PHREG	*b*'*	-.713	-.665	-.636	-.636	-.628	-.626	-.625	-.625
	c'*	-.571	-.512	-.515	-.499	-.503	-.500	-.502	-.501
	*c**	-.495	-.465	-.471	-.461	-.462	-.461	-.463	-.462
PH/REG	*ab*'*	.003	-.001	.001	-.003	.001	.000	.000	.000
	c-c*'*	.076	.047	.045	.038	.041	.039	.039	.039
	ab'-(c*-c*')*	-.073	-.048	-.044	-.041	-.040	-.039	-.039	-.039
Both dropout and study duration censoring									
PHREG	*b*'*	-.744	-.674	-.639	-.639	-.630	-.627	-.626	-.626
	c'*	-.626	-.513	-.517	-.501	-.501	-.501	-.502	-.501
	*c**	-.546	-.466	-.472	-.465	-.463	-.465	-.466	-.464
PH/REG	*ab*'*	.001	-.002	.002	-.003	.000	.000	.000	.000
	c-c*'*	.081	.046	.045	.036	.038	.036	.037	.036
	ab'-(c*-c*')*	-.080	-.048	-.043	-.039	-.038	-.037	-.037	-.037

Table B11

Power and Type I error for 1-wave mediator (no mediation) using LIFEREG

Method									
Sample Size		20	50	100	200	500	1000	2000	5000
REG	*a*	.293	.407	.465	.501	.524	.521	.522	.527
No censoring									
LIFEREG	*b*'	.368	.448	.488	.522	.522	.530	.522	.525
	c'	.258	.404	.671	.904	.999	1.000	1.000	1.000
	c	.245	.403	.645	.869	.991	1.000	1.000	1.000
LIFE/REG	*a* & *b*'	.043	.042	.055	.052	.046	.051	.044	.052
	mediation	.010	.014	.023	.025	.025	.026	.022	.024
Dropout censoring									
LIFEREG	*b*'	.337	.433	.465	.508	.527	.529	.524	.525
	c'	.211	.307	.518	.796	.987	1.000	1.000	1.000
	c	.197	.309	.510	.772	.978	.999	1.000	1.000
LIFE/REG	*a* & *b*'	.045	.046	.053	.048	.051	.050	.046	.052
	mediation	.008	.010	.018	.023	.024	.028	.023	.025
Study duration censoring									
LIFEREG	*b*'	.313	.427	.462	.511	.524	.527	.524	.523
	c'	.152	.288	.522	.790	.991	1.000	1.000	1.000
	c	.126	.274	.499	.771	.987	1.000	1.000	1.000
LIFE/REG	*a* & *b*'	.034	.040	.050	.052	.048	.048	.046	.050
	mediation	.006	.009	.019	.023	.025	.024	.024	.024
Both dropout and study duration censoring									
LIFEREG	*b*'	.289	.408	.451	.498	.522	.527	.524	.524
	c'	.146	.231	.413	.693	.969	.999	1.000	1.000
	c	.118	.219	.408	.676	.960	.999	1.000	1.000
LIFE/REG	*a* & *b*'	.035	.043	.053	.049	.047	.048	.046	.051
	mediation	.003	.008	.016	.019	.024	.027	.024	.023

Table B12

Power and Type I error for 1-wave mediator (no mediation) using PHREG

Method									
Sample Size		20	50	100	200	500	1000	2000	5000
No censoring									
PHREG	*b**'	.311	.435	.482	.520	.522	.529	.521	.524
	*c**'	.173	.350	.636	.897	.999	1.000	1.000	1.000
	*c**	.154	.337	.599	.849	.989	1.000	1.000	1.000
PH/REG	*a* & *b**'	.030	.037	.053	.051	.046	.050	.043	.052
	mediation	.005	.011	.019	.022	.017	.018	.012	.011
Dropout censoring									
PHREG	*b**'	.262	.414	.460	.507	.527	.528	.523	.523
	*c**'	.127	.257	.484	.776	.988	1.000	1.000	1.000
	*c**	.106	.250	.466	.744	.975	1.000	1.000	1.000
PH/REG	*a* & *b**'	.029	.040	.051	.049	.051	.049	.045	.051
	mediation	.002	.008	.017	.019	.019	.021	.012	.011
Study duration censoring									
PHREG	*b**'	.284	.420	.461	.511	.523	.527	.523	.523
	*c**'	.132	.277	.516	.786	.991	1.000	1.000	1.000
	*c**	.115	.264	.498	.767	.986	1.000	1.000	1.000
PH/REG	*a* & *b**'	.028	.038	.049	.051	.048	.048	.046	.050
	mediation	.003	.008	.018	.022	.022	.020	.015	.013
Both dropout and study duration censoring									
PHREG	*b**'	.240	.398	.452	.496	.522	.527	.524	.524
	*c**'	.110	.220	.407	.690	.970	.999	1.000	1.000
	*c**	.096	.207	.404	.670	.960	.998	1.000	1.000
PH/REG	*a* & *b**'	.026	.039	.054	.048	.047	.048	.046	.051
	mediation	.002	.007	.015	.017	.021	.023	.015	.013

Table B13

Mean values for parameter estimates for 2-wave mediator (partial mediation) using LIFEREG

Method									
Sample Size		20	50	100	200	500	1000	2000	5000
REG	*a*	.296	.300	.302	.301	.300	.301	.300	.300
No censoring									
	b'	.501	.501	.501	.500	.500	.501	.500	.500
LIFEREG	*c*'	.196	.200	.200	.200	.200	.199	.200	.200
	c	.341	.350	.351	.351	.349	.349	.350	.351
	ab'	.147	.151	.151	.150	.150	.150	.150	.150
LIFE/REG	*c-c*'	.146	.150	.151	.152	.150	.150	.150	.151
	ab'-(*c-c*')	.002	.001	.000	-.002	.001	.000	.000	.000
Dropout censoring									
	b'	.510	.506	.503	.500	.499	.501	.500	.500
LIFEREG	*c*'	.194	.198	.198	.198	.199	.199	.200	.200
	c	.333	.341	.342	.341	.341	.341	.342	.342
	ab'	.151	.152	.152	.150	.150	.150	.150	.150
LIFE/REG	*c-c*'	.139	.143	.145	.143	.142	.142	.142	.142
	ab'-(*c-c*')	.012	.010	.007	.008	.008	.008	.008	.008
Study duration censoring									
	b'	.533	.514	.508	.502	.501	.501	.501	.501
LIFEREG	*c*'	.200	.201	.200	.200	.200	.200	.201	.200
	c	.330	.325	.322	.322	.319	.320	.320	.320
	ab'	.157	.155	.153	.151	.150	.151	.150	.150
LIFE/REG	*c-c*'	.130	.125	.122	.122	.119	.120	.119	.120
	ab'-(*c-c*')	.028	.030	.031	.029	.031	.031	.031	.030
Both dropout and study duration censoring									
	b'	.538	.516	.509	.503	.501	.501	.501	.500
LIFEREG	*c*'	.205	.200	.199	.199	.199	.200	.200	.200
	c	.341	.329	.325	.325	.323	.323	.323	.323
	ab'	.161	.155	.153	.151	.150	.151	.150	.150
LIFE/REG	*c-c*'	.137	.128	.126	.125	.123	.123	.123	.123
	ab'-(*c-c*')	.024	.027	.027	.026	.027	.027	.027	.027

Note. Mean parameter values: *a*=.30, *b*'=.50, *c*'=.20, *c*=.35, *ab*'=.15, *c-c*'=.15, *ab*'-(*c-c*')=0.

Table B14

Mean values for parameter estimates for 2-wave mediator (partial mediation) using PHREG

Method	Sample Size	20	50	100	200	500	1000	2000	5000
No censoring									
PHREG	$b^{*\prime}$	-1.466	-1.321	-1.283	-1.263	-1.255	-1.255	-1.251	-1.252
	$c^{*\prime}$	-.578	-.528	-.511	-.505	-.501	-.499	-.501	-.501
	c^{*}	-.625	-.585	-.570	-.565	-.556	-.555	-.555	-.556
PH/REG	$ab^{*\prime}$	-.434	-.398	-.386	-.380	-.377	-.377	-.375	-.376
	$c^{*}-c^{*\prime}$	-.047	-.057	-.060	-.061	-.055	-.057	-.054	-.055
	$ab^{*\prime}-(c^{*}-c^{*\prime})$	-.386	-.341	-.327	-.320	-.322	-.320	-.320	-.321
Dropout censoring									
PHREG	$b^{*\prime}$	-1.599	-1.344	-1.294	-1.268	-1.256	-1.255	-1.253	-1.251
	$c^{*\prime}$	-.646	-.530	-.509	-.503	-.501	-.498	-.500	-.501
	c^{*}	-.706	-.617	-.604	-.598	-.591	-.589	-.590	-.590
PH/REG	$ab^{*\prime}$	-.477	-.405	-.390	-.381	-.377	-.377	-.375	-.376
	$c^{*}-c^{*\prime}$	-.060	-.087	-.096	-.095	-.090	-.091	-.089	-.089
	$ab^{*\prime}-(c^{*}-c^{*\prime})$	-.417	-.318	-.294	-.287	-.286	-.286	-.286	-.287
Study duration censoring									
PHREG	$b^{*\prime}$	-1.532	-1.339	-1.290	-1.266	-1.256	-1.254	-1.252	-1.252
	$c^{*\prime}$	-.574	-.523	-.508	-.503	-.501	-.499	-.501	-.501
	c^{*}	-.649	-.629	-.619	-.620	-.614	-.615	-.616	-.616
PH/REG	$ab^{*\prime}$	-.455	-.404	-.388	-.381	-.377	-.377	-.375	-.376
	$c^{*}-c^{*\prime}$	-.074	-.106	-.111	-.116	-.113	-.116	-.114	-.116
	$ab^{*\prime}-(c^{*}-c^{*\prime})$	-.381	-.298	-.277	-.264	-.263	-.261	-.261	-.260
Both dropout and study duration censoring									
PHREG	$b^{*\prime}$	-1.654	-1.355	-1.298	-1.269	-1.257	-1.255	-1.252	-1.251
	$c^{*\prime}$	-.649	-.527	-.506	-.504	-.500	-.500	-.501	-.501
	c^{*}	-.718	-.643	-.632	-.633	-.627	-.628	-.629	-.630
PH/REG	$ab^{*\prime}$	-.494	-.408	-.391	-.382	-.377	-.377	-.375	-.376
	$c^{*}-c^{*\prime}$	-.069	-.116	-.126	-.130	-.128	-.129	-.128	-.129
	$ab^{*\prime}-(c^{*}-c^{*\prime})$	-.425	-.292	-.265	-.252	-.249	-.248	-.247	-.247

Table B15

Power to detect non-zero effects for 2-wave mediator (partial mediation) using LIFEREG

Method									
Sample Size		20	50	100	200	500	1000	2000	5000
REG	*a*	.244	.529	.745	.903	.997	1.000	1.000	1.000
No censoring									
LIFEREG	*b*'	.644	.832	.924	.982	1.000	1.000	1.000	1.000
	c'	.246	.411	.663	.915	1.000	1.000	1.000	1.000
	c	.352	.576	.815	.960	.999	1.000	1.000	1.000
LIFE/REG	*a* & *b*'	.151	.437	.689	.887	.997	1.000	1.000	1.000
	mediation	.084	.307	.587	.839	.983	.999	1.000	1.000
Dropout censoring									
LIFEREG	*b*'	.553	.774	.882	.953	.999	1.000	1.000	1.000
	c'	.216	.309	.500	.789	.993	1.000	1.000	1.000
	c	.274	.471	.717	.915	.999	1.000	1.000	1.000
LIFE/REG	*a* & *b*'	.129	.402	.657	.859	.996	1.000	1.000	1.000
	mediation	.051	.235	.508	.782	.972	.996	.999	1.000
Study duration censoring									
LIFEREG	*b*'	.554	.781	.880	.952	1.000	1.000	1.000	1.000
	c'	.184	.315	.521	.801	.992	1.000	1.000	1.000
	c	.212	.458	.729	.942	1.000	1.000	1.000	1.000
LIFE/REG	*a* & *b*'	.127	.408	.656	.860	.997	1.000	1.000	1.000
	mediation	.046	.244	.517	.806	.976	.998	1.000	1.000
Both dropout and study duration censoring									
LIFEREG	*b*'	.491	.736	.849	.928	.997	1.000	1.000	1.000
	c'	.181	.257	.420	.709	.976	1.000	1.000	1.000
	c	.181	.389	.643	.894	.997	1.000	1.000	1.000
LIFE/REG	*a* & *b*'	.115	.385	.635	.837	.994	1.000	1.000	1.000
	mediation	.031	.201	.454	.750	.967	.995	1.000	1.000

Table B16

Power to detect non-zero effects for 2-wave mediator (partial mediation) using PHREG

Method									
Sample Size		20	50	100	200	500	1000	2000	5000
No censoring									
PHREG	*b*'*	.558	.811	.918	.982	1.000	1.000	1.000	1.000
	c'*	.163	.362	.631	.903	1.000	1.000	1.000	1.000
	*c**	.221	.487	.767	.953	1.000	1.000	1.000	1.000
PH/REG	*a* & *b*'*	.129	.424	.686	.887	.997	1.000	1.000	1.000
	mediation	.041	.218	.440	.623	.669	.686	.664	.630
Dropout censoring									
PHREG	*b*'*	.438	.745	.871	.949	.999	1.000	1.000	1.000
	c'*	.126	.256	.463	.770	.992	1.000	1.000	1.000
	*c**	.164	.390	.662	.901	.999	1.000	1.000	1.000
PH/REG	*a* & *b*'*	.101	.386	.649	.856	.996	1.000	1.000	1.000
	mediation	.023	.166	.392	.618	.757	.779	.799	.814
Study duration censoring									
PHREG	*b*'*	.485	.770	.878	.951	1.000	1.000	1.000	1.000
	c'*	.136	.291	.511	.796	.992	1.000	1.000	1.000
	*c**	.185	.438	.718	.938	1.000	1.000	1.000	1.000
PH/REG	*a* & *b*'*	.108	.404	.655	.859	.997	1.000	1.000	1.000
	mediation	.030	.199	.440	.687	.814	.859	.900	.939
Both dropout and study duration censoring									
PHREG	*b*'*	.401	.720	.844	.928	.997	1.000	1.000	1.000
	c'*	.117	.231	.406	.702	.976	1.000	1.000	1.000
	*c**	.149	.364	.623	.888	.997	1.000	1.000	1.000
PH/REG	*a* & *b*'*	.090	.377	.630	.837	.994	1.000	1.000	1.000
	mediation	.019	.162	.384	.644	.830	.878	.922	.968

Table B17

Mean values for parameter estimates for 2-wave mediator (complete mediation) using LIFEREG

Method									
Sample Size		20	50	100	200	500	1000	2000	5000
REG	*a*	.296	.300	.302	.301	.301	.301	.299	.300
No censoring									
	b'	.498	.502	.501	.499	.500	.501	.500	.501
LIFEREG	*c*'	-.002	-.001	-.001	-.001	.000	-.001	.000	.000
	c	.142	.151	.151	.151	.150	.149	.150	.151
	ab'	.146	.151	.151	.150	.150	.150	.150	.150
LIFE/REG	*c*-*c*'	.144	.151	.152	.152	.150	.150	.150	.150
	ab'-(*c*-*c*')	.002	.000	-.001	-.001	.000	.001	.000	.000
Dropout censoring									
	b'	.507	.506	.503	.499	.500	.501	.500	.500
LIFEREG	*c*'	-.006	-.002	-.003	-.002	.000	-.001	.000	.000
	c	.138	.145	.146	.144	.146	.145	.146	.146
	ab'	.151	.152	.152	.150	.150	.151	.150	.150
LIFE/REG	*c*-*c*'	.143	.148	.149	.146	.147	.146	.146	.146
	ab'-(*c*-*c*')	.008	.005	.003	.004	.003	.004	.004	.004
Study duration censoring									
	b'	.529	.514	.508	.503	.501	.501	.501	.501
LIFEREG	*c*'	-.006	-.002	-.002	-.002	-.001	-.001	.000	.000
	c	.139	.140	.136	.136	.135	.135	.135	.136
	ab'	.156	.155	.154	.152	.150	.151	.150	.150
LIFE/REG	*c*-*c*'	.145	.142	.138	.137	.135	.135	.135	.135
	ab'-(*c*-*c*')	.011	.013	.016	.014	.015	.015	.015	.015
Both dropout and study duration censoring									
	b'	.534	.515	.510	.503	.502	.501	.501	.500
LIFEREG	*c*'	-.003	-.004	-.004	-.002	-.001	-.001	.000	.000
	c	.145	.138	.137	.136	.137	.136	.137	.137
	ab'	.160	.156	.154	.152	.151	.151	.150	.150
LIFE/REG	*c*-*c*'	.148	.142	.140	.139	.138	.137	.137	.137
	ab'-(*c*-*c*')	.012	.013	.014	.013	.013	.014	.013	.013

Note. Mean parameter values: *a*=.30, *b*'=.50, *c*'=0, *c*=.15, *ab*'=.15, *c*-*c*'=.15, *ab*'-(*c*-*c*')=0.

Table B18

Mean values for parameter estimates for 2-wave mediator (complete mediation) using PHREG

Method									
Sample Size		20	50	100	200	500	1000	2000	5000
No censoring									
	*b**'	-1.455	-1.324	-1.284	-1.263	-1.256	-1.255	-1.251	-1.252
PHREG	*c**'	.000	.002	.003	.002	.001	.003	.000	.000
	*c**	-.251	-.238	-.231	-.230	-.225	-.224	-.225	-.226
	*ab**'	-.428	-.399	-.387	-.381	-.377	-.377	-.375	-.376
PH/REG	*c**-*c**'	-.250	-.240	-.234	-.232	-.226	-.227	-.225	-.225
	*ab**'-(*c**-*c**')	-.177	-.159	-.154	-.149	-.151	-.150	-.150	-.150
Dropout censoring									
	*b**'	-1.584	-1.345	-1.295	-1.267	-1.256	-1.255	-1.253	-1.252
PHREG	*c**'	.007	.007	.007	.005	.001	.003	.000	-.001
	*c**	-.274	-.249	-.245	-.242	-.242	-.239	-.241	-.241
	*ab**'	-.475	-.405	-.391	-.382	-.377	-.377	-.375	-.376
PH/REG	*c**-*c**'	-.281	-.256	-.253	-.248	-.243	-.242	-.241	-.241
	*ab**'-(*c**-*c**')	-.194	-.149	-.139	-.134	-.134	-.135	-.134	-.135
Study duration censoring									
	*b**'	-1.515	-1.338	-1.291	-1.268	-1.257	-1.254	-1.252	-1.252
PHREG	*c**'	.015	.007	.005	.005	.002	.002	-.001	-.001
	*c**	-.262	-.260	-.253	-.253	-.251	-.251	-.253	-.254
	*ab**'	-.447	-.404	-.390	-.382	-.377	-.377	-.375	-.376
PH/REG	*c**-*c**'	-.277	-.267	-.258	-.258	-.253	-.254	-.252	-.253
	*ab**'-(*c**-*c**')	-.170	-.137	-.132	-.124	-.125	-.123	-.123	-.123
Both dropout and study duration censoring									
	*b**'	-1.630	-1.354	-1.301	-1.270	-1.258	-1.255	-1.253	-1.252
PHREG	*c**'	.009	.012	.008	.005	.002	.002	.000	-.001
	*c**	-.283	-.261	-.258	-.258	-.258	-.257	-.259	-.260
	*ab**'	-.488	-.409	-.393	-.382	-.378	-.377	-.375	-.376
PH/REG	*c**-*c**'	-.292	-.273	-.266	-.264	-.260	-.259	-.258	-.259
	*ab**'-(*c**-*c**')	-.195	-.136	-.127	-.119	-.118	-.118	-.117	-.117

Table B19

Power to detect non-zero effects for 2-wave mediator (complete mediation) using LIFEREG

Method									
Sample Size		20	50	100	200	500	1000	2000	5000
REG	*a*	.244	.524	.745	.907	.997	1.000	1.000	1.000
No censoring									
LIFEREG	*b*'	.639	.831	.925	.982	1.000	1.000	1.000	1.000
	c'	.123	.076	.056	.059	.052	.051	.052	.052
	c	.177	.221	.291	.439	.641	.792	.916	.982
LIFE/REG	*a* & *b*'	.150	.432	.690	.892	.997	1.000	1.000	1.000
	mediation	.044	.144	.253	.423	.641	.792	.916	.982
Dropout censoring									
LIFEREG	*b*'	.549	.774	.885	.952	.999	1.000	1.000	1.000
	c'	.129	.075	.064	.052	.052	.054	.055	.054
	c	.156	.170	.236	.351	.581	.740	.882	.966
LIFE/REG	*a* & *b*'	.128	.399	.660	.865	.996	1.000	1.000	1.000
	mediation	.033	.107	.200	.336	.579	.740	.882	.966
Study duration censoring									
LIFEREG	*b*'	.555	.775	.881	.954	1.000	1.000	1.000	1.000
	c'	.099	.056	.055	.051	.048	.057	.055	.051
	c	.084	.144	.219	.356	.585	.758	.887	.968
LIFE/REG	*a* & *b*'	.127	.403	.656	.865	.997	1.000	1.000	1.000
	mediation	.024	.097	.192	.342	.584	.757	.887	.968
Both dropout and study duration censoring									
LIFEREG	*b*'	.486	.735	.852	.930	.996	1.000	1.000	1.000
	c'	.108	.061	.060	.054	.048	.056	.057	.052
	c	.083	.118	.191	.306	.535	.707	.859	.956
LIFE/REG	*a* & *b*'	.113	.380	.636	.845	.993	1.000	1.000	1.000
	mediation	.015	.072	.162	.291	.534	.706	.858	.955

Table B20

Power to detect non-zero effects for 2-wave mediator (complete mediation) using PHREG

Method									
Sample Size		20	50	100	200	500	1000	2000	5000
No censoring									
PHREG	*b**'	.555	.813	.916	.981	1.000	1.000	1.000	1.000
	*c**'	.075	.064	.052	.055	.050	.052	.052	.052
	*c**	.089	.145	.228	.379	.607	.776	.908	.980
PH/REG	*a* & *b**'	.129	.424	.683	.891	.997	1.000	1.000	1.000
	mediation	.022	.101	.200	.363	.602	.773	.906	.980
Dropout censoring									
PHREG	*b**'	.434	.753	.876	.951	.999	1.000	1.000	1.000
	*c**'	.065	.058	.058	.050	.050	.051	.053	.053
	*c**	.068	.115	.190	.311	.548	.721	.873	.965
PH/REG	*a* & *b**'	.098	.388	.652	.864	.996	1.000	1.000	1.000
	mediation	.012	.070	.161	.298	.544	.717	.872	.964
Study duration censoring									
PHREG	*b**'	.488	.767	.881	.955	1.000	1.000	1.000	1.000
	*c**'	.064	.049	.053	.049	.048	.056	.055	.051
	*c**	.071	.131	.209	.346	.579	.753	.885	.967
PH/REG	*a* & *b**'	.110	.400	.657	.866	.997	1.000	1.000	1.000
	mediation	.018	.087	.182	.329	.574	.749	.883	.967
Both dropout and study duration censoring									
PHREG	*b**'	.400	.719	.849	.930	.996	1.000	1.000	1.000
	*c**'	.063	.054	.058	.051	.047	.055	.058	.052
	*c**	.062	.107	.178	.295	.525	.702	.857	.955
PH/REG	*a* & *b**'	.092	.373	.633	.844	.993	1.000	1.000	1.000
	mediation	.009	.063	.149	.279	.521	.699	.855	.954

Table B21

Mean values for parameter estimates for 2-wave mediator (no mediation) using LIFEREG

Method									
Sample Size		20	50	100	200	500	1000	2000	5000
REG	*a*	.246	.250	.252	.251	.250	.251	.249	.250
No censoring									
LIFEREG	*b*'	.249	.252	.251	.250	.250	.251	.250	.251
	c'	.198	.200	.200	.199	.200	.199	.200	.200
	c	.195	.202	.201	.201	.199	.199	.200	.200
LIFE/REG	*ab*'	-.004	.001	.001	.000	.000	.000	.000	.000
	c-*c*'	-.003	.002	.001	.002	-.001	.000	.000	.000
	ab'-(*c*-*c*')	-.001	.000	-.001	-.002	.001	.000	.000	.000
Dropout censoring									
LIFEREG	*b*'	.252	.254	.252	.250	.249	.251	.250	.250
	c'	.195	.199	.198	.198	.200	.199	.200	.200
	c	.189	.198	.198	.196	.197	.197	.198	.198
LIFE/REG	*ab*'	-.002	.001	.001	.000	-.001	.000	.000	.000
	c-*c*'	-.006	-.001	-.001	-.001	-.003	-.002	-.002	-.002
	ab'-(*c*-*c*')	.005	.003	.001	.002	.002	.003	.002	.002
Study duration censoring									
LIFEREG	*b*'	.262	.259	.254	.252	.250	.251	.250	.250
	c'	.207	.202	.203	.200	.200	.200	.201	.200
	c	.193	.194	.193	.191	.190	.190	.191	.191
LIFE/REG	*ab*'	-.004	.002	.000	.001	-.001	.000	.000	.000
	c-*c*'	-.015	-.008	-.010	-.009	-.011	-.010	-.010	-.010
	ab'-(*c*-*c*')	.010	.010	.011	.009	.010	.010	.010	.010
Both dropout and study duration censoring									
LIFEREG	*b*'	.265	.259	.255	.252	.250	.251	.250	.250
	c'	.213	.202	.202	.200	.200	.200	.201	.200
	c	.199	.193	.193	.192	.190	.191	.192	.192
LIFE/REG	*ab*'	-.002	.001	.000	.000	-.001	.000	.000	.000
	c-*c*'	-.015	-.008	-.009	-.008	-.010	-.009	-.009	-.009
	ab'-(*c*-*c*')	.013	.010	.009	.008	.009	.009	.009	.009

Note. Mean parameter values: *a*=.25, *b*'=.25, *c*'=.20, *c*=.20, *ab*'=0, *c*-*c*'=0, *ab*'-(*c*-*c*')=0.

Table B22

Mean values for parameter estimates for 2-wave mediator (no mediation) using PHREG

Method									
Sample Size		20	50	100	200	500	1000	2000	5000
No censoring									
PHREG	*b**'	-.721	-.665	-.643	-.630	-.628	-.629	-.625	-.627
	*c**'	-.588	-.532	-.514	-.504	-.502	-.499	-.501	-.501
	*c**	-.433	-.422	-.409	-.406	-.400	-.400	-.401	-.402
PH/REG	*ab**'	.011	-.003	-.001	-.001	.001	-.001	.000	.000
	*c**-*c**'	.154	.110	.105	.099	.102	.099	.099	.099
	*ab**'-(*c**-*c**')	-.143	-.113	-.106	-.099	-.101	-.100	-.099	-.099
Dropout censoring									
PHREG	*b**'	-.782	-.675	-.649	-.634	-.628	-.628	-.626	-.626
	*c**'	-.630	-.536	-.513	-.503	-.502	-.499	-.501	-.501
	*c**	-.460	-.429	-.419	-.415	-.412	-.411	-.413	-.413
PH/REG	*ab**'	.005	-.001	-.002	-.001	.001	.000	.000	.000
	*c**-*c**'	.171	.107	.094	.087	.090	.088	.088	.088
	*ab**'-(*c**-*c**')	-.165	-.108	-.096	-.088	-.089	-.089	-.088	-.088
Study duration censoring									
PHREG	*b**'	-.752	-.678	-.648	-.634	-.628	-.628	-.625	-.626
	*c**'	-.588	-.524	-.514	-.502	-.502	-.500	-.502	-.501
	*c**	-.438	-.434	-.428	-.426	-.423	-.424	-.426	-.426
PH/REG	*ab**'	.012	-.005	-.001	-.001	.001	.000	.000	.000
	*c**-*c**'	.150	.090	.086	.076	.080	.076	.076	.075
	*ab**'-(*c**-*c**')	-.137	-.094	-.087	-.078	-.078	-.076	-.076	-.075
Both dropout and study duration censoring									
PHREG	*b**'	-.803	-.685	-.651	-.635	-.629	-.628	-.626	-.626
	*c**'	-.651	-.529	-.513	-.503	-.501	-.500	-.502	-.501
	*c**	-.478	-.436	-.431	-.430	-.426	-.427	-.430	-.430
PH/REG	*ab**'	.006	-.003	-.001	-.001	.001	.000	.000	.000
	*c**-*c**'	.173	.093	.082	.073	.075	.073	.072	.071
	*ab**'-(*c**-*c**')	-.166	-.096	-.083	-.074	-.074	-.073	-.072	-.072

Table B23

Type I error for 2-wave mediator (no mediation) using LIFEREG

Method									
Sample Size		20	50	100	200	500	1000	2000	5000
REG	*a*	.285	.405	.465	.496	.524	.530	.526	.522
No censoring									
LIFEREG	*b*'	.379	.450	.490	.518	.532	.525	.527	.524
	c'	.250	.401	.649	.899	.998	1.000	1.000	1.000
	c	.235	.368	.559	.803	.964	.995	1.000	1.000
LIFE/REG	*a* & *b*'	.044	.046	.053	.050	.056	.055	.053	.047
	mediation	.007	.015	.019	.025	.029	.030	.025	.022
Dropout censoring									
LIFEREG	*b*'	.339	.426	.468	.508	.529	.525	.522	.526
	c'	.222	.308	.494	.783	.990	1.000	1.000	1.000
	c	.191	.289	.436	.692	.937	.987	1.000	1.000
LIFE/REG	*a* & *b*'	.048	.048	.052	.052	.053	.055	.048	.048
	mediation	.008	.009	.016	.021	.025	.027	.021	.022
Study duration censoring									
LIFEREG	*b*'	.326	.422	.468	.505	.529	.527	.527	.524
	c'	.176	.299	.506	.781	.987	1.000	1.000	1.000
	c	.124	.252	.437	.704	.949	.994	1.000	1.000
LIFE/REG	*a* & *b*'	.038	.044	.052	.050	.053	.057	.054	.046
	mediation	.003	.009	.016	.019	.023	.024	.020	.017
Both dropout and study duration censoring									
LIFEREG	*b*'	.299	.402	.450	.493	.526	.525	.524	.524
	c'	.176	.241	.412	.683	.967	.999	1.000	1.000
	c	.107	.200	.343	.614	.919	.988	1.000	1.000
LIFE/REG	*a* & *b*'	.041	.043	.051	.050	.051	.055	.050	.046
	mediation	.004	.008	.014	.017	.025	.024	.018	.017

Table B24

Type I error for 2-wave mediator (no mediation) using PHREG

Method									
Sample Size		20	50	100	200	500	1000	2000	5000
No censoring									
PHREG	*b**'	.316	.435	.487	.518	.531	.524	.528	.524
	*c**'	.177	.358	.624	.890	.998	1.000	1.000	1.000
	*c**	.143	.295	.502	.767	.963	.995	1.000	1.000
PH/REG	*a* & *b**'	.034	.043	.052	.049	.055	.054	.054	.047
	mediation	.003	.010	.010	.017	.016	.012	.009	.009
Dropout censoring									
PHREG	*b**'	.264	.409	.463	.501	.529	.525	.521	.526
	*c**'	.135	.264	.467	.767	.988	1.000	1.000	1.000
	*c**	.105	.220	.384	.663	.930	.986	1.000	1.000
PH/REG	*a* & *b**'	.034	.046	.052	.049	.053	.055	.047	.048
	mediation	.003	.006	.009	.014	.015	.013	.008	.011
Study duration censoring									
PHREG	*b**'	.276	.416	.464	.504	.529	.527	.528	.524
	*c**'	.143	.284	.495	.776	.987	1.000	1.000	1.000
	*c**	.116	.243	.425	.699	.945	.993	1.000	1.000
PH/REG	*a* & *b**'	.029	.042	.050	.050	.053	.057	.054	.046
	mediation	.002	.008	.012	.014	.016	.014	.011	.013
Both dropout and study duration censoring									
PHREG	*b**'	.236	.394	.445	.492	.525	.524	.525	.524
	*c**'	.121	.223	.402	.678	.967	.999	1.000	1.000
	*c**	.092	.186	.342	.604	.915	.987	1.000	1.000
PH/REG	*a* & *b**'	.030	.043	.049	.049	.050	.054	.051	.046
	mediation	.003	.006	.011	.014	.016	.014	.010	.012
